# Factors Reducing the Use of a Persuasive mHealth App and How to Mitigate Them: Thematic Analysis

**DOI:** 10.2196/40579

**Published:** 2023-06-26

**Authors:** Markku Kekkonen, Eveliina Korkiakangas, Jaana Laitinen, Harri Oinas-Kukkonen

**Affiliations:** 1 Oulu Advanced Research on Service and Information Systems Faculty of Information Technology and Electrical Engineering University of Oulu Oulu Finland; 2 Finnish Institute of Occupational Health Oulu Finland

**Keywords:** mobile phone, mobile health, mHealth, Persuasive Systems Design, behavior change, thematic analysis, microentrepreneurs, randomized controlled trial

## Abstract

**Background:**

Studies on which persuasive features may work for different users in health contexts are rare. The participants in this study were microentrepreneurs. We built a persuasive mobile app to help them to recover from work. Representatives of this target group tend to be very busy due to work, which was reflected in their use of the app during the randomized controlled trial intervention. Microentrepreneurs also often have dual roles; they are professionals in their line of work as well as entrepreneurs managing their own business, which may add to their workload.

**Objective:**

This study aimed to present users’ views on the factors that hinder their use of the mobile health app that we developed and how these factors could be mitigated.

**Methods:**

We interviewed 59 users and conducted both data-driven and theory-driven analyses on the interviews.

**Results:**

Factors reducing app use could be divided into 3 categories: use context (problem domain–related issues, eg, the lack of time due to work), user context (user-related issues, eg, concurrent use of other apps), and technology context (technology-related issues, eg, bugs and usability). Due to the nature of the participants’ entrepreneurship, which often interferes with personal life, it became clear that designs targeting similar target groups should avoid steep learning curves and should be easy (quick) to use.

**Conclusions:**

Personalized tunneling—guiding the user through a system via personalized solutions—could help similar target groups with similar issues better engage with and keep using health apps because of the easy learning curve. When developing health apps for interventions, background theories should not be interpreted too strictly. Applying theory in practice may require rethinking approaches for adaptation as technology has evolved rapidly and continues to evolve.

**Trial Registration:**

ClinicalTrials.gov NCT03648593; https://clinicaltrials.gov/ct2/show/NCT03648593

## Introduction

### Overview

Health care can be improved by cost-effective solutions with the help of modern health information technologies [1]. In particular, the development of mobile health (mHealth) apps can provide cost-efficient health interventions for a wide range of users, although user preferences may vary considerably [2]. Designing health apps for diverse target groups may seem to be an insurmountable challenge, as stakeholders may have different views on what is important. However, these views are not necessarily mutually exclusive [3]. Even when designing health apps with special attention to a target group, it is likely that some software features will not be used as much as anticipated by the designers [4].

Engaging users to continue to use health apps is challenging. Persuasive technologies could lessen the challenge, especially if the characteristics of different users are addressed [5]. The persuasive features in digital health interventions supporting users can increase user adherence [6]. According to Fogg [7], persuasion is “an attempt to change attitudes or behaviors or both (without using coercion or deception).”

Designers can use persuasive technologies to motivate people to change their health behavior toward a preferred behavior [8]. Therefore, the use of persuasive technologies to support health behavior change could be beneficial. However, although studies on persuasion extend back to at least 2000 years, persuasion is still not fully understood [8]. Human psychology is complex, and designers may experience challenges when designing persuasive systems [8]. Despite software designers’ best efforts and use of persuasive technologies, getting users to stay active remains a challenge, especially because there are numerous health apps available.

Although research has been conducted on adherence and engagement with digital health apps and interventions [9,10], studies on factors that reduce the use of mHealth apps from users’ perspectives are rare, especially users’ views on how to mitigate these factors.

Designers often add features to an “implementation wish list,” but such features must be justified. Therefore, designers should know which features are persuasive for which target groups. However, this can be challenging if information on the target group is scarce.

Although there are studies on which features or persuasive categories may work for general users in a health context [11] or which persuasive features have been used in specific types of health apps [12], it is more difficult to determine what works for specific groups. Thus, more research is needed on the persuasive features for a variety of target groups.

This study aimed to increase the knowledge on the factors that hinder or reduce the use of persuasive mHealth apps and how these factors can be mitigated. To achieve this, we conducted a data-driven thematic analysis based on interviews (N=59) conducted with users of an mHealth app. In addition, to understand how to avoid pitfalls, we conducted a theory-driven thematic analysis of the interviews. The novelty of this study lies in the persuasion event analysis (data driven) regarding factors reducing the use of the app and the persuasive software feature analysis (theory driven) on ways to mitigate or even improve these factors.

The interviews were conducted as part of an 8-week randomized controlled trial that aimed to help microentrepreneurs recover from work and job strain. The mHealth app for the intervention trial was developed in collaboration with a multidisciplinary research consortium. The Persuasive Systems Design (PSD) [13] model was used as the framework for designing the persuasive technology features within the app. Self-determination theory (SDT) [14] was used as the theoretical background for behavior change, and the transtheoretical model (TTM) [15] was adopted for “Stages of Change”–driven goal setting within the app.

### Research Question

To gain more knowledge on the topic, we wanted to understand why some users stopped using the app. We also wanted to learn how user engagement could be increased for similar target groups and persuasive mHealth apps.

Therefore, the following two research questions guided this paper:

1. Research question 1: What were the factors hindering or reducing the use of the app?
2. Research question 2: How can the persuasive side of the mHealth app be improved using PSD considering the aforementioned factors?

### Background

#### Microentrepreneurs as the Target Group

In EU countries in 2014, small and medium-sized companies (SMEs) accounted for 99.8% of all enterprises (in the nonfinancial sector), employing approximately 90 million people [16]. The threshold for defining SMEs in the European Union is up to 250 employees and <€50 million (US $54 million) in financial turnover, with smaller firms usually having fewer than 50 employees and <€10 million (US $11 million) in turnover [17].

In 2014, about 93% of the SMEs in EU countries were microenterprises [16], which are small companies with <10 employees and €2 million (US $2.1 million) in financial turnover [17]. Therefore, in the European Union, microenterprises and microentrepreneurs are vital for national economies. Moreover, in 2016, from 70% to 95% of all firms in all countries were microenterprises, with a large share of those being enterprises with no employees, thus running solely by the microentrepreneurs themselves [18].

Entrepreneurship involves many factors that can cause high workloads, and there is an obvious need to promote work recovery. However, there have been few interventions targeting work recovery in microenterprises [19]. According to Voltmer et al [20], the health of an entrepreneur influences the development of a successful enterprise.

Entrepreneurs are at an increased risk of overexertion [20] because of high responsibilities and demands at work, stress, excessive working hours, fatigue, and sleeping problems [21]. Entrepreneurs also have difficulties balancing work and leisure time [22-26]. Thus, they might benefit from interventions to cope with these professional demands and stress as well as promote healthy behavior patterns [20].

Small businesses are a suitable target group for health promotion [21], but tailored, simple, and low-cost actions are required [27]. Effective recovery from work requires healthy lifestyles [23], including sufficient physical activity, healthy dietary habits [28-31], and stress and time management [23]. Planning beforehand, controlling overtime, having work flexibility, having social contacts, and exercising regularly are all strategies that can help entrepreneurs maintain good health [23].

#### Underlying Theories for the Developed System

The app used SDT [14] as the theoretical background for users’ behavior change process, thus allowing users to navigate within the system relatively freely. This approach gave the users the freedom to choose any and all content material, tasks, or tools within the app or to choose none. The app also provided relevant and nonjudgmental feedback for the users.

Although users’ self-determination was strongly emphasized, there were minor limitations on user actions within the app owing to development requirements. Before gaining access to the health problem domains, the users had to proceed through 52 baseline questions about their current health behavior, although the questions could be left unanswered. Similarly, in the beginning of each health problem domain module, the users had to proceed through content-specific introductory material once. However, this could also be skipped by pressing the “forward” or “home” buttons.

TTM includes 6 stages of change [15]; however, to avoid complicated goal setting structures for the app, we used an adaptation of TTM. Thus, each module contained 3 goal setting categories based on TTM: *think and observe (contemplation and preparation)*, *act and do (action)*, and *maintenance (maintenance)*. *Precontemplation* was excluded from the app, as people in that stage would not be ready to proceed toward change and thus could not be engaged. *Termination* was also excluded because people in that final stage would have no need for the app. Each TTM-based goal setting category contained interactive tasks in all health problem domain modules. After choosing a health domain, users could also choose which stage they wanted, and they were not assigned to any specific goal-setting category by the system. Regarding the first 2 categories, the tasks could be completed either in minutes or within a day or 2.

**Figure 1:**
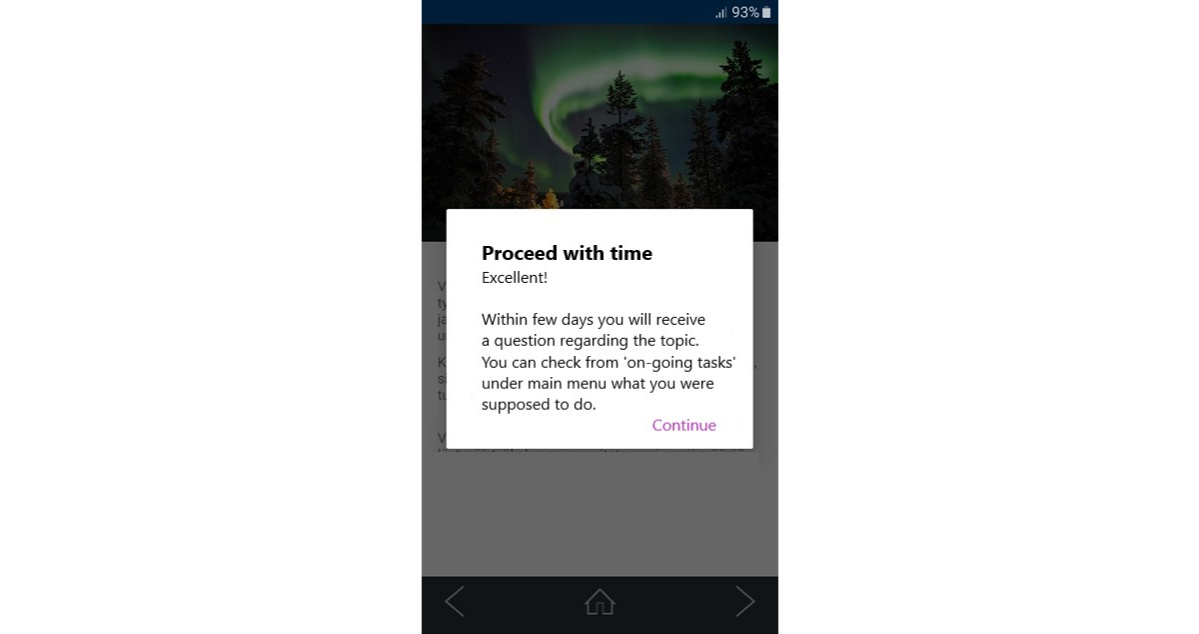
The user has triggered a longer task, which will require a few days to complete, depending on when the user wants to complete it.

The tasks in the *Maintenance* category were supposed to be completed over a longer period, for example, within 10 days. Reminders in the form of push notifications were sent to users who had chosen tasks that required a longer time to complete. Figure 1 shows an edited screenshot of the app (textual content originally in Finnish but translated for this paper).

TTM has been criticized as inappropriate for some behavior change interventions [32]; however, in this case, we feel that the adaptation provided a clear and easy way for users to follow their situations and progress as they worked toward their personal behavioral goals.

#### PSD Model

PSD [13] is a model for persuasive software design with design principles for persuasive system functionalities and content. The PSD model offers postulates for describing and evaluating persuasive systems and ways to analyze the persuasion context. There are 4 categories of persuasive principles in the PSD model: primary task support (eg, rehearsal), dialogue support (eg, praise), system credibility support (eg, authority), and social support (eg, normative influence) [13].

The use context, user context, and technology context are the key factors in analyzing persuasive events. The use context includes features or factors arising from the problem domain, such as health behavior. The user context refers to people’s individual differences, including their interests, needs, goals, motivations, lifestyles, and other cultural factors. Regarding the technology context, the strengths, weaknesses, risks, and opportunities of different platforms, apps, and features should be considered [13].

#### Postulates

According to the first postulate of the PSD model, information technology is not neutral but rather is “always on,” and thus persuasion can be an ongoing process instead of a single act [13]. Therefore, persuasion and persuasive systems require active participation (using the system) from the users, but the system also has to be there for the users for persuasion to happen.

The second postulate of the PSD model emphasizes commitment for cognitive consistency [13]. On one hand, it means that persuasive systems should support and facilitate commitments. On the other hand, users may become committed to performing the target behavior by the support provided by the persuasive system, which naturally means that they should also use the system to achieve this. In terms of SDT, it could be thought that the users should “know” and perform the right actions to achieve their goals, and persuasive systems can support this.

When considering the third postulate, which deals with direct and indirect (or a combination of both) persuasion strategies, it may be difficult to determine which strategy to use. For example, direct persuasion might be more enduring than indirect persuasion strategies. However, an indirect strategy could be better for individuals who are in a hurry or in the event of information overflow via the persuasive system. Therefore, it is necessary to know the audience—the target users who are going to use the app.

As stated in the fourth postulate of the PSD model, persuasion is often incremental, and therefore persuasive systems can enable users to proceed toward the target behavior through a series of incremental steps [13]. By using a persuasive system, the users should be encouraged to take small steps at the beginning and then take larger steps toward the target behavior over the course of the use process. TTM is suitable for incremental persuasion, as it is inherently divided into different stages, with the first stage focused on preparing users to achieve their personal behavior change goals.

The fifth postulate stresses system transparency, whereas the sixth postulate emphasizes unobtrusiveness [13]. If the system is biased with false information or disturbs the users, the outcomes in terms of behavioral change may be less than desirable. The mHealth app was backed up by a trusted national institute (the Finnish Institute of Occupational Health) and was designed to be unobtrusive. However, the experiences of some users may have varied due to bugs, specifically push notifications triggering at less-than-ideal times.

The seventh postulate of the PSD model, regarding the usefulness and ease of use of a persuasive system, indicates that useless systems or ones that are difficult to use are not that persuasive [13]. If the software quality of a system is poor or lacking, there is a high possibility that the system will not be used for a long time or continuously by the users. However, the situation is not specific to persuasive systems; rather, it applies to all information systems. Therefore, poor usability or bugs might reduce the use and thus the overall persuasiveness of any system.

### The System

#### Overview

As the app was developed with the help of the PSD model, we analyzed and then selected the persuasive features to be used together with the research consortium. A workshop was held within the consortium at the beginning of the whole project, where principal investigators and researchers eventually chose the initial features based on reflections regarding the target group, previous experiences from similar research settings, and the trial context.

Furthermore, persuasive features were discussed with representatives of the target group in a series of focus group meetings and workshops [33]. During consortium meetings, the final set of features was eventually formed through discussions on what could support the target group, with background theories taken into consideration.

#### PSD Features

The persuasive features included in the app were based on the following PSD principles [13]: self-monitoring, rehearsal, praise, reminders, suggestion, liking, trustworthiness, and social comparison (Table 1).

**Table 1:** Principles that were implemented in the system.

System support category	Principle	Example from the app
Primary task	Self-monitoring	Step counter
Primary task	Rehearsal	Cyclic nutrition rehearsal tool
Dialogue	Praise	Positive feedback
Dialogue	Reminders	Push notifications
Dialogue	Suggestion	Pop-up giving a suggestion for behavior
Dialogue	Liking	Visually attractive pictures
Credibility	Trustworthiness	Evidence-based information
Social	Social comparison	Module proposition based on all users’ answers

#### Health Domains

**Figure 2:**
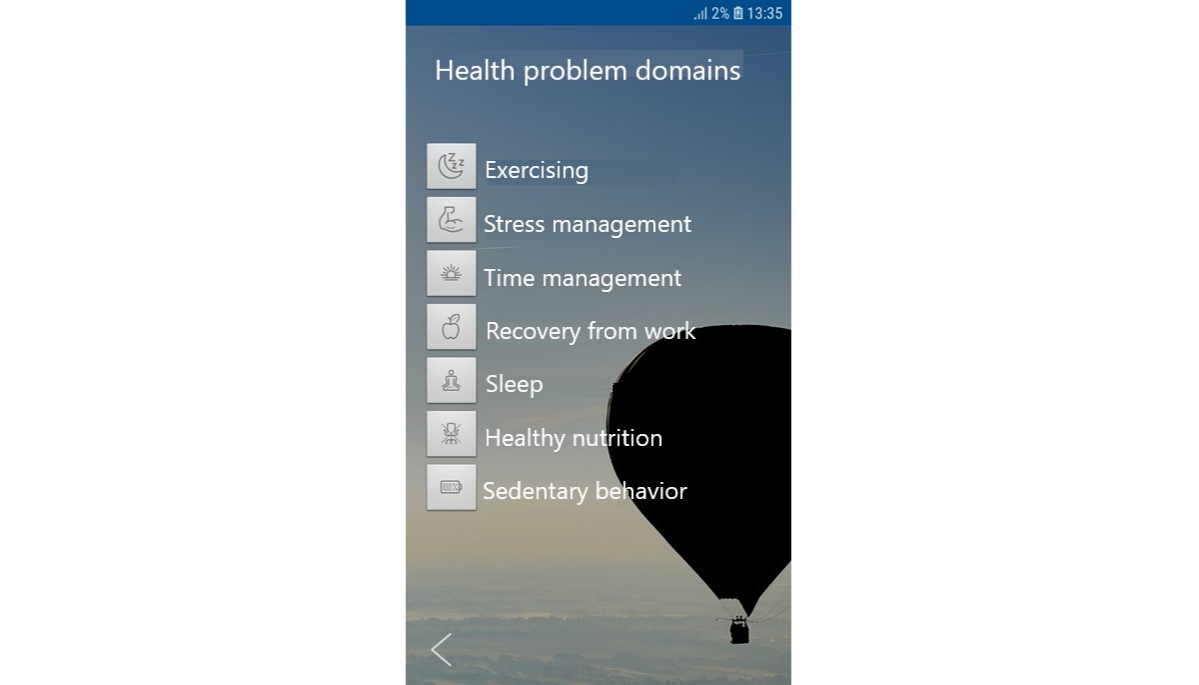
Health problem domains in the app.

Another paper by Laitinen et al [34] describes the study protocol of the randomized controlled trial, including the hypothesis behind the following health problem domains in the app: (1) exercising (physical activity), (2) stress management, (3) time management (efficient working hours), (4) recovery from work, (5) sleep, (6) healthy nutrition (dietary behavior), and (7) sedentary behavior (excessive sitting). In addition, the work by Tiitinen et al [35] was important to our choice of health domains. Figure 2 presents an edited screenshot of the app (textual content originally in Finnish but translated for this paper).

The results regarding the primary and secondary outcomes for the randomized controlled trial will be published in a separate paper in the future.

#### App

We developed the system for the Android smartphone platform as a native app, which means that it was implemented using a compiled language (Java in this case) instead of web technologies [36]. Given the number of different Android smartphone devices available from various manufacturers and the differences within the Android operating system versions, the development process might have been less resource consuming using web technologies. Nevertheless, the native app approach could support use even without a network connection, thus enabling its use in remote locations, which we felt was an advantage over web-based apps.

Some software features implemented were more complicated in terms of programming, such as the step counter, whereas some were relatively simple in design. We used library packs provided by Android for the step counter; however, it took a relatively long time to test the functionality while adjusting the step counter. Thus, for resource reasons, we do not recommend adding complex tools to research purpose apps, as they are easily available elsewhere, and programming one from scratch (even with library packs) may require considerable time and resources.

The functionalities were designed to be simple and easy to implement. For example, we added a pop-up for certain intervals that provided relevant tips (per health module) to users. Similar to other tools, we strived for a simple yet efficient design, keeping in mind that we were designing a research app, not a finished and polished commercial product.

Another example of a simple design was the sit-stand reminder tool with an alarm. Although it would be possible to use the native alarm clock of one’s phone, it required relatively few resources (programming hours) to add one in the app. Thus, the users could use the tool easily within the app, as they were already committed to the trial. It would have served no purpose to ask users to find and install simple tools on their phones.

#### Lessons Learned

The lower limit of the Android version for using the app was 4.4, with no upper limit. The latest Android version available at the time of the intervention was 8.1. In hindsight, it would have been better to start development with the latest version, as the development took more than a year, and thus versions 4.4 up to 6 were already becoming outdated when the trial began. Too much variety in the Android versions increased opportunities for bugs because different Android versions, for example, use different libraries, and all differences between the versions had to be taken into consideration before the release of the app.

We should also have anticipated that many of our target users would have the latest smartphones and thus the latest Android versions. In Finland, entrepreneurs can deduct the cost of work phones from their taxes. However, we also wanted to be fair and include earlier Android versions because not all microentrepreneurs can afford new phones. Indeed, self-employed entrepreneurs may live from “paycheck to paycheck,” only able to pay themselves salary depending on their sales or the number of customers in a given month.

## Methods

### Recruitment

Our research consortium recruited microentrepreneurs for the intervention via various means, such as email, and >1200 eligible participants were enrolled to participate in the randomized controlled trial. The recruitment process has been described in detail in another study [37].

### The Trial

The enrolled participants were randomized into 2 groups: one for the actual intervention (613 participants) and another for control (612 participants). The control group was granted access to the same app with the same features at a later date than the intervention group. All participants were instructed to freely choose any of the health domains in their preferred order, and the app offered them the information and tools to reach their individual goals. In addition, they could use reflective questions in the app to determine their current situation regarding the health domains. We also informed them that they could freely perform any tasks within their preferred categories or just do them partially and they could always return to the tasks later. The participants were not compensated for participating in the intervention.

Although the intervention period was 8 weeks, the users could continue their use freely even after that period ended. The trial was conducted in Finland, and the participants eligible for the intervention had to live in Finland during the intervention and understand Finnish. Using an Android-based smartphone was essential and compulsory for participating in the intervention as well as being an actual microentrepreneur with fewer than 10 employees and financial revenue of €2 million (US $2.1 million).

All the interviewees in this study comprised of the intervention group. The full protocol of the trial is reported in another paper [34].

### Data Collection

The interviews were based on semistructured questions in Finnish with 2 different emphases: the system (health behavior change, user, and use experiences; question set 1; Multimedia Appendix 1) and recovery from work (microentrepreneurs’ health and ways of living and app use for recovery; question set 2; Multimedia Appendix 2). The responses to the questions and discussions during the interviews were used to form the data sets: data set 1 (the system) and data set 2 (recovery from work). Although the angles varied in the interviews, the topics overlapped, and thus both data sets included discussions on similar matters.

We decided to use both data sets for this study as they complement each other, which leads to a more complete picture of the phenomenon under study. The questions for both interviews were piloted with the representative users before the trial.

Participants from the intervention group who had given their consent to be contacted were randomized into 2 lists for the interviews, and each of the 2 research teams responsible for the interviews received one list. The participants were contacted an equal number of times to obtain their final consent for the interviews and to schedule them. The first data set consisted of the interviews of 29 participants, whereas the second data set consisted of the interviews of 30 participants. Thus, a total of 59 interviews were conducted in this study.

The interviews were mainly conducted using Skype, a voice-over IP software program. Telephone calls were offered as an alternative in the event that participants could not use Skype for some reason. The recorded interviews were transcribed manually by third-party professionals. The contents of the interviews were not altered during the transcription process.

### Ethics Approval, Informed Consent, and Participation

All the participants provided informed consent and were informed of their ability to opt out. The participants were interviewed as part of the randomized controlled trial study, which was approved by the Ethics Committee of the Finnish Institute of Occupational Health in November 2017 (#5/2017).

### Research Methodology

**Figure 3:**
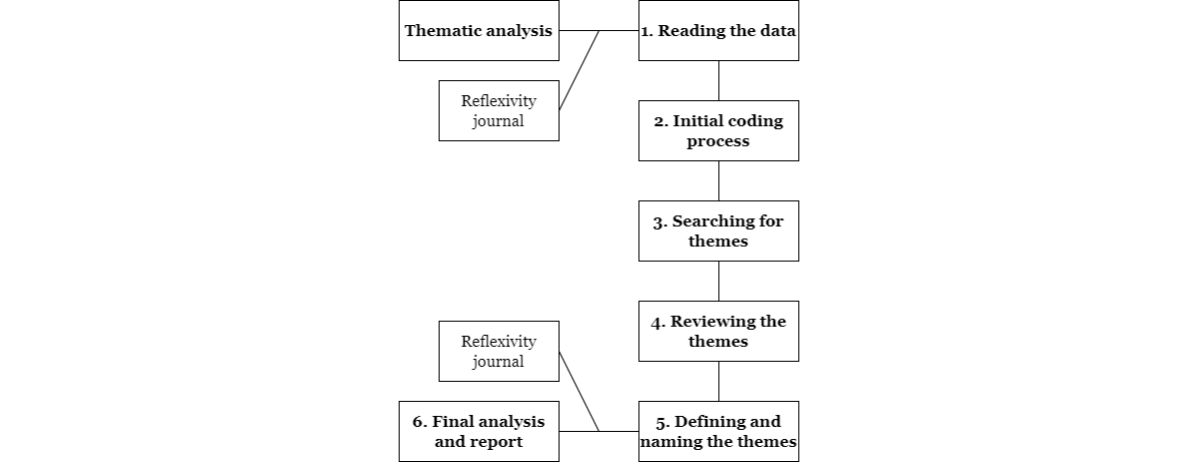
The 6 phases of thematic analysis.

We decided to use thematic analysis as the research method. According to Braun and Clarke [38], it is “a method for identifying, analyzing, and reporting patterns (themes) within data. It minimally organizes and describes your data set in (rich) detail.” There were 6 phases in the thematic analysis [38,39] (Figure 3).

Our data corpus consisted of the interviews, with each interview being a data item. Data extracts were individually coded chunks of data that were identified within and extracted from data items [38].

To accurately answer our 2 research questions, we performed 2 analyses. For the first analysis (persuasion event context analysis), we chose an inductive (data-driven) approach to identify the factors that hinder or reduce the use of the app. Thus, we did not attempt to fit the data based on preexisting frames or preconceptions. Direct implications for theory are not the priority in a data-driven qualitative data analysis and may not even be required. However, the theoretical implications cannot be fully ignored [38].

For the second analysis (PSD analysis), we used a deductive (theory-driven) approach, allowing us to compare the interviewees’ perspectives against the PSD framework model to see how we could mitigate the factors according to the interviewees’ views. The results of both analyses are further discussed in the *Discussion* section.

### Data Analysis Process

#### First Phase

The transcribed interviews were carefully read 3 times to get familiar with the data [38,39]. A reflexivity journal [39] was initiated at this point in the form of memos and notes [38]. The journal was updated constantly throughout the analysis process. The first phase was similar in both the analyses.

#### Second Phase—Persuasion Event Analysis

Computer software can be helpful for the coding process [40]. Therefore, the transcribed interviews were imported into NVivo (QSR International), a qualitative analysis program. We generated initial codes from the data [38,39], which were divided into 2 deductive categories: (1) technical reasons reducing the use of the app and (2) other reasons reducing the use. Combining deductive and inductive approaches in thematic analysis is not uncommon and may be used when necessary [39,41,42].

#### Second Phase—PSD Analysis

The transcribed documents were imported into NVivo for coding. Initial codes were created and divided into four deductive theme categories according to PSD: (1) primary task support, (2) dialogue support, (3) system credibility support, and (4) social support. A theory, framework, or model can be used for creating deductive categories when performing a deductive analysis [38].

#### Third Phase—Persuasion Event Analysis

In this phase, we started to search for themes [38,39] from the codes in the initial coding categories. We identified 10 potential themes from the codes. These themes were moved into separate theme nodes (with work-in-progress names) in NVivo, deriving the following candidate versions of the themes: other apps, busy, content, Hawthorne, format, disappointment, usability, stress, life, and bugs.

#### Third Phase—PSD Analysis

Next, we formed subthemes for each main theme (PSD category) from the PSD model using category-related PSD principles [13] as subthemes. The codes were moved into equivalent or suitable subtheme nodes that best matched the codes. In this phase, we found that some codes seemed to overlap with the PSD principles; therefore, these codes were placed into ≥2 subtheme nodes at the same time for later decision-making in the next phase.

#### Fourth Phase—Persuasion Event Analysis

In this phase, we reviewed and refined the themes carefully [38,39]. First, we identified 2 general main themes (parent nodes in NVivo) from the candidate themes: “technology-related” reasons and “user-related” reasons that reduced and hindered the use of the app. The technology-related themes were linked to the app itself, whereas user-related themes were naturally linked to the users themselves.

However, we noticed that some subthemes found in the inductive analysis did not fit either theme. Thus, we added a third main theme: “use-related” reasons. As part of the review process, unnecessary and overlapping codes were deleted [38,39]. To tie the inductive analysis into the theoretical commitment [38], each refined subtheme was placed under the matching main theme (by switching to a deductive approach) with the help of the PSD definition of the persuasion event context regarding use, users, and technology. However, the subthemes remained relatively broad. Hence, we divided each subtheme into smaller nodes to highlight these issues in more detail.

#### Fourth Phase—PSD Analysis

We then determined the final subtheme placement for overlapping codes and deleted duplicates from other subthemes. We also noticed that some subthemes were either empty or the data we had for them were not rich enough for certain results, which may often happen [38]. In such cases, themes were removed from the analysis, but not before we returned to the raw data [39].

#### Fifth Phase–Persuasion Event Analysis

In this phase, the final themes were defined. We adapted the main themes under matching persuasion event context themes: use context, user context, and technology context [13]. Subthemes, including smaller nodes within the subthemes, were also given their final names [38,39] while trying to avoid refining the themes for too long. A reflexivity journal (memos and notes) was maintained during the entire process, which involved writing about the analysis of the themes.

#### Fifth Phase—PSD Analysis

We went through the data and coding 2 times in this phase to ensure that we could develop credible results for the final analysis [39,40]. Similar to the persuasion event analysis, we completed the final analysis of the themes with the help of the reflexivity journal.

#### Sixth Phase

The last phase consisted of producing final versions of both the analyses and reports [38,39] using direct quotes. The reflexivity journal provided support in writing the analyses and report, which are presented as results in this paper. The entire research process (Figure 3) was time-consuming, but every phase was needed to conduct a thematic analysis [38]. Although thematic analysis can yield interesting qualitative results, the analysis process can sometimes be long and complex.

## Results

### Persuasion Event—Use Context

#### Overview

The lack of time due to work was the most common subtheme, and 71% (42/59) of the interviewees indicated that they were spending a lot of time working. We also found it to be linked with other subthemes. For example, the interviewees expressed that if the use was complex, they did not want to spend time on the learning curve, thus abandoning the app in the worst-case scenario.

A similar example of the lack of time reflected in other subthemes was that many interviewees felt that they were too busy to read the instructions or module introductions properly. Therefore, they may have concluded that the app was malfunctioning. However, the backend system that logged data from user interactions with the system offered a different view (although there were bugs in the system, not all “malfunctions” were bugs according to the log data). We think that some interviewees assumed that the app was malfunctioning when it may have been that they did not have time to learn to use the app properly (or to read the instructions).

The idea that technology is not working could also stem from technostress. In fact, technostress was a major subtheme within the interviews, as 29% (17/59) of the interviewees expressed feelings of stress due to the use of technology.

Furthermore, content-related issues were very common, and 59% (35/59) of the interviewees expressed having some issues with the content. It should be noted that the content of the app was somewhat extensive, and thus it is not surprising that this would arise as one of the top reasons for reducing app use. The results for each subtheme regarding use context themes are presented in Table 2.

**Table 2:** Use context themes found in the analysis.

Use context themes		Interviewees (data set 1; n=29), n (%)	Interviewees (data set 2; n=30), n (%)	All interviewees (N=59), n (%)
**Lack of time due to work**		24 (100)	18 (100)	42 (100)
	Busy at work	24 (100)	18 (100)	42 (100)
	Excessive working hours	3 (12)	5 (28)	8 (19)
**Content-related issues**		22 (100)	13 (100)	35 (100)
	Information overflow	10 (45)	7 (54)	17 (49)
	Need for advanced content	13 (59)	3 (23)	16 (46)
	Contents not suitable	5 (23)	4 (31)	9 (26)
	Too much textual content	3 (14)	3 (23)	6 (17)
**Technostress**		11 (100)	6 (100)	17 (100)
	Invasive technology	5 (45)	4 (67)	9 (53)
	Stressed from using the app	3 (27)	2 (33)	5 (29)
	System too complex	3 (27)	0 (0)	3 (18)

#### Lack of Time Due to Work

In one way or another, 71% (42/59) of the interviewees expressed being busy due to work. Furthermore, 19% (8/42) of them even reported working 7-day weeks or 15-to-16-hour days. Excessive working habits can affect life in several ways, for example, leaving little or no time to spend with family. Therefore, it is unsurprising that using the app might not be the first thing that the interviewees did when they had little time for themselves. In some cases, they even felt guilty for being too busy to use the app:

I feel guilty, because using the app would not have taken that much time, no need to inspect everything for hours, so it would have fit [into daily routines] and I could have done something every day. I was so busy then, but now when I’m not that busy anymore, I have actually used the app more.

Interviewee #3

Many interviewees expressed that work came first, as they felt that they were responsible not only for themselves but also for their families and for their employees and their employees’ families. They seemed to be interested in changing their poor health choices to healthier ones, but they often neglected themselves and their own health because they were busy. For 71% (42/59) of the interviewees, being busy due to work clearly reduced the use of the app, as they prioritized working over the app or even over everything else:

It was four p.m., after which I used to work for four more hours, and I didn’t have time for anything else. My social life was suffering, and I spent the weekends at work. When there [from the app] came those reminders, I was at work. I just always ignored the reminders, because I never had any time to use it [the app].

Interviewee #55

The interviewees reported that during the intervention period they had a lot of work, it was their best seasonal time for working, or that they had to prepare for the coming season. The interviewees seemed to have more work available than they could complete within “normal” office hours. This resulted in tiredness due to long workdays, which left no time for anything else:

At the beginning of spring, I was having this contract job that had been going on already for few months. It required me to drive tens of kilometers every morning, after which I did a long day and drove back. I was very tired, and I thought that this must stop, or I’ll stop being an entrepreneur. I was so tired, and I had no time for anything else [than work].

Interviewee #19

It was not just the actual work that caused the interviewees to be busy. They also expended considerable effort in obtaining contracts, jobs, or orders as well as in other work-related tasks, such as financial management and replying to customers. As microentrepreneurs, the interviewees also managed their own companies and possibly even had employees to manage, which led to more working hours. They also reported continuing working at home after they had left the workplace for the day:

I don’t have time to get everything done during the day, so I work at evenings and nights too. I might go to bed at the same time as my kid, but then I wake up during the small hours to work, or I work at midnight.

Interviewee #32

#### Content-Related Issues

Overall, 27% (16/59) of the interviewees believed that the information provided by the app was general, with little new information to offer. Furthermore, 10% (6/59) of the interviewees thought that there was too much textual information to read:

If that wall of text is even necessary, and this felt somewhat like lectures in the app, I really don’t know which kind of people even need that.

Interviewee #5

The information content was clearly problematic for some of the interviewees. They complained that they were unsure if the app was meant for them. For example, there were some tasks they felt that they could not complete, such as talking to colleagues (when one was working alone). Given the extensive amount of content, it was inevitable that some aspects of the content might be problematic for some users—for example, if the user did not perform office work with excessive sitting but was advised to stand up periodically. Better personalization could have solved this issue.

However, this was not a problem for all of the respondents, as they reported that they went through only the parts of the content that they needed. Even so, the wide scope of the app content presented a problem for 29% (17/59) of the interviewees in the form of information overflow, as they were unsure which health problem module or tasks and tools to pick:

It takes a lot of time [to use] and last winter [time of the use] I was often very tired, so I found it hard to concentrate on these things here, because there are so much content and different modules.

Interviewee #15

#### Technostress

We noticed that when the users were busy because of work, they were also stressed because of work. Smartphones were seen as one of the tools for working, which caused stress. During busy periods, the interviewees thought that their phones were ringing “all the time,” and they also felt that they had to answer the phone when a customer was calling. Adding technostress to the equation of excessive working and being tired seemed to increase their perceived stress. Simply having to use a smartphone was named as a stressor in addition to receiving push notifications (reminders) from the phone.

Technostress, the inability to adapt to rapidly deployed new technologies, may have physical consequences for users, such as headaches, restlessness, or fatigue [43], or increase stress hormone production [44]. Overall, 8% (5/59) of the interviewees reported that using the app caused technostress for them:

It was stressful and that I was supposed to be a slave to the phone even more, when I was thinking that I don’t want to check my phone all the time. It was the third day when I uninstalled the app.

Interviewee #19

### Persuasion Event—User Context

#### Overview

In the user context, concurrent use of wearables or another app was the most common theme, as reported by 37% (22/59) of the interviewees (Table 3). It should be noted that while some users decided to use the alternative that best suited their needs, others chose to continue this concurrent use until the end of the intervention period. Other themes stemming from the users (which most people could relate to) were disadvantageous life situations, including health conditions, unfulfilled expectations (eg, vague “need” for something that is lacking), and different coaching preferences (eg, personal trainer instead of app).

**Table 3:** User context themes found in the analysis.

User context themes		Interviewees (data set 1; n=29), n (%)	Interviewees (data set 2; n=30), n (%)	All interviewees (N=59), n (%)
**Concurrent use of wearables or another app**		10 (100)	7 (100)	17 (100)
	Using another application at the same time	6 (60)	5 (71)	11 (65)
	Using wearables at the same time	7 (70)	3 (43)	10 (59)
**Disadvantageous life situation**		1 (100)	12 (100)	13 (100)
	Medical condition	0 (0)	6 (50)	6 (43)
	Mental health issues	0 (0)	4 (33)	4 (31)
	Changes in everyday life	1 (100)	1 (8)	2 (15)
	Family-related issues	0 (0)	3 (25)	3 (25)
**Expectations unfulfilled**		6 (100)	5 (100)	11 (100)
	Expectations did not match with reality	2 (33)	3 (60)	5 (45)
	Lacking features	2 (33)	2 (40)	4 (36)
	Lacking something	2 (33)	0 (0)	2 (18)
**Different coaching preference**		4 (100)	6 (100)	10 (100)
	Face-to-face preferred	1 (25)	3 (50)	4 (40)
	Nondigital self-help preferred	3 (75)	2 (33)	5 (50)
	Medical measurements preferred	1 (25)	0 (0)	1 (10)
	Peer support groups preferred	0 (0)	1 (17)	1 (10)

#### Concurrent Use of Wearables or Another App

Overall, 29% (17/59) of the interviewees used wearables or another app during the intervention period, often concurrently with the persuasive mHealth app used in the trial. Wearables (eg, smartwatches) were popular as self-monitoring tools, for example, for measuring the user’s steps or heartbeat. One reason for using wearables was that people might leave their phones on their desks while walking to the printer. In contrast, wearable devices could be carried easily without any extra effort:

I don’t carry my phone with me all the time, and the app assumed that everyone would carry her or his phone everywhere. I have a smartwatch, which I use for measuring my steps and pretty much for everything else, too.

Interviewee #5

Users who were not interested in reading or who disliked the coaching approach of the app seemed to find wearables or simple sport apps better suited for their needs. They seemed to be mainly interested in measuring different health-related aspects, such as heartbeat or sleep, and were less interested in being coached. However, they could use the trial app concurrently with the wearables or another app:

I have recently installed another app, which I use for following what I eat, but otherwise I don’t have anything else related to health in my phone. Oh, but wait, I do have an activity band too, from which I get data into my phone, and then there is the app of yours. I have noticed that these are helpful for checking things out.

Interviewee #16

#### Disadvantageous Life Situation

People tend to experience different situations in their lives, which could hinder or decrease the use of any app. The microentrepreneurs interviewed were no different, as they were troubled by loud neighbors or experienced insomnia, insecurities regarding their business, health issues (or their relatives had health issues), etc. For some people, combining entrepreneurship and family life can be difficult, as both might require a considerable amount of time. As the app was dealing with health problem domains, it was unsurprising that 17% (10/59) of the interviewees reported experiencing either mental or physical health conditions, which reduced their use of the app:

It is probably because of my condition, as I can’t concentrate on anything in the kitchen. That section, “plan your meals” in the app, well that planning thing, as well as putting it into practice, is difficult for me because of my condition.

Interviewee #50

#### Expectations Unfulfilled

Overall, 19% (11/59) of the interviewees had great expectations for the app but were disappointed in practice. In other words, their expectations did not necessarily match the reality of using the app. If users have predefined needs, and if they think that they cannot fulfill those needs with the help of the app used, they will surely be disappointed. This will inevitably reduce their use of any app.

The interviewees found it difficult to point out exactly what it was that they were missing, but they mentioned issues such as networking (with other entrepreneurs), peer support, and various automated measurement functions:

I didn’t find what I was looking for, although clarifying what I wanted is difficult, but I thought that it could have automatically offered what I needed. I cannot really put it into words, just a thought in my head, but it should have measured me automatically during the workday, like how much I am sitting or how stressed I am, or other stuff like that. Pretty tough demands and so on.

[Interviewee #20]

#### Different Coaching Preference

Apparently, not all users knew what they were enrolling in, although the intervention was advertised as the use of an evidence-based coaching app for behavioral changes. For example, 3% (2/59) of the interviewees complained about the chosen coaching approach, stating that they would have preferred to see a health care professional face-to-face. Two other interviewees resorted to hiring someone to help them (eg, a personal trainer), which led them to abandoning the app:

I have glanced at the contents, but it didn’t inspire me that much, because I had a chance for this hired personal guidance face-to-face. In my opinion, an app can’t compete with humans yet, and I managed to get expert guidance otherwise.

Interviewee #34

### Persuasion Event—Technology Context

#### Overview

Of the 59 interviewees, 30 (51%) complained of technical issues. It should be noted that being busy at work (no time to learn to use the app or read instructions) could have affected this perception. We do not disagree with their views (as views tend to be subjective experiences), but we do conclude from log data that not everything reported as a technical error was one. However, if the interviewees felt that there were technical issues, then it does not matter whether they were real. Better usability (considering both the use and user contexts) could have solved this issue, at least to some level.

Usability issues were common factors hindering the use, and 37% (22/59) of the interviewees reported such issues. Usability was also partially tied to content-related issues. As a background theory, SDT affected the usability and content of the app. When designing the app, we assumed that people would make the “right” choices most of the time (from the given options).

Some users perceived it difficult to choose a health problem domain from the options while also having to choose the proper stage of change when wanting to perform tasks from the modules. In addition, 17% (10/59) of the interviewees did not like the platform that was used (native Android app; Table 4).

**Table 4:** Technology context themes found in the analysis.

Technology context themes		Interviewees (data set 1; n=29), n (%)	Interviewees (data set 2; n=30), n (%)	All interviewees (N=59), n (%)
**Technical issues**		20 (100)	10 (100)	30 (100)
	Major bugs	5 (25)	3 (30)	8 (27)
	Minor bugs	16 (80)	7 (70)	23 (77)
**Usability issues**		14 (100)	8 (100)	22 (100)
	Difficult learning curve	10 (71)	5 (62)	15 (68)
	Complexity issues	5 (36)	4 (50)	9 (41)
	Memorability	1 (7)	1 (12)	2 (9)
**Disfavored platform**		3 (100)	7 (100)	10 (100)
	Different format preferred	2 (67)	6 (86)	8 (80)
	Different operating system preferred	1 (33)	1 (14)	2 (20)

#### Technical Issues

In the interviews, many users complained about numerous but mostly minor issues with the app (eg, incorrect font size and screen scale). One crucial bug related to persuasiveness was that the weekly push notification reminders did not work for all users. Overall, 25% (15/59) of the interviewees reported that they did not receive weekly reminders, or that when interacting with the weekly reminder and trying to answer the questionnaire, they could not submit the answer:

That recovery statistic reminder or something like that, there was a bug, since even after two weeks when trying to input the answer, the program replied instantly that I had updated the answer already in that week, and I should try again later.

Interviewee #54

On one hand, if they did not receive the weekly reminder, participants reported that they forgot to use the app or the feature with the malfunctioning reminder. On the other hand, when they received a malfunctioning weekly reminder, it also decreased the persuasiveness and use of the app owing to user frustration or disappointment because of the bug.

For 8% (5/59) of the interviewees, the push notifications occasionally malfunctioned and looped the weekly reminder or task reminders unnecessarily. In a rare case, a large question set (supposed to be triggered at the end of the intervention period via push notification) was looped, which eventually led the interviewee to abandon the app:

That set of questions was long and so I tried to proceed from it, answered the questions and accepted them, ok. This kind of app should be intuitive, so nothing is left hanging. I think I made it to the end and continued from there, where there were these tasks and picked few of them to start with. The next time I used the app, it wanted me to do the question set again, and I did not want to do that. It offered it to me at least three or four times.

Interviewee #26

In addition, 7% (4/59) of the interviewees reported that the app either froze or crashed on their smartphones occasionally, but otherwise the bugs mostly hindered rather than prevented use. Nevertheless, any bug, whether minor or major, might reduce the persuasiveness and use of any app. On the one hand, users might wonder if it is worth continuing to use an app that does not seem to work properly—there are many alternatives in the commercial market. On the other hand, bugs might be something that more experienced users have become used to, at least to some extent, as one interviewee expressed:

Oh well, it must be because usually all of these [health apps] don’t necessary work, so I’ve gotten used to it that these just happen to have these [bugs].

Interviewee #3

#### Usability Issues

All 22 (37%) interviewees in this theme were either unsure about how they should have used the app or felt that the app was too complex. They complained that the learning curve was too high and that there were no clear instructions on how to use the app (or that they could not find the instructions):

When going through the app, I thought that there would be instructions on how to use it, how it works, what is the idea behind it, but I didn’t find anything like that. A month later, I think, I found instructions from somewhere, which explained a little.

Interviewee #7

It should be mentioned that when logging into the app for the first time, there were instructions on how to use the app and the concept behind it, but some users skipped the introduction. The same introductory text was also available under the main menu. In addition, the instructions on how tasks work were available each time the user chose a task.

Nevertheless, when users felt that instructions were lacking, it reduced their use because they were unsure of how to use the app. At a general level, some people may be irritated by excessive explanations and instructions, whereas others may quit using apps because of a lack of clear and plentiful instructions. The interviews also showed that another usability flaw from the users’ viewpoint was the lack of an option to check which tasks had already been performed:

I want to see my progress, so in that sense, for example in tasks there is no list of what I have already done, or anything like that, where I could check on how the task went.

Interviewee #3

#### Disfavored Platform

Overall, 7% (4/59) of the interviewees did not like using the app on the smartphone and would have preferred alternatives. One of them reported that because of a medical condition, a keyboard and a mouse would have been a better option, as handling a touchscreen on a smartphone was painful. Another would have preferred a radio broadcast (podcast) for guidance rather than a smartphone app. Activity bracelets and smart watches have also been mentioned as a preferred platform because they have better sensors and offer automatic measurement. Furthermore, 3% (2/59) of the interviewees complained that because only an Android version was available, they had to use it with their secondary phones, as they mainly used iPhones. This evidently decreased their use of the app:

I use iPhone, but I have one Android phone, into which I installed this app, because there was not an iPhone version available. I do not normally use an Android phone. Because of that, I haven’t used the app very much.

Interviewee 53

It should be mentioned that dozens of iPhone users enrolled in the intervention, although it was clearly advertised that the app was available only for Android smartphones. The enrollment web form included a specific question about whether the users had an Android phone. If a potential participant answered that they did not have or use an Android smartphone, the enrollment did not continue. Apparently, these people either answered incorrectly to continue or did not read the question properly.

Moreover, our helpdesk was approached several times via email by iPhone users complaining about the lack of an iOS version. It is therefore possible that several participants switched from Android phones to iPhones between the enrollment phase and the start of the trial.

### PSD Analysis—Persuasive Categories and Principles

#### Overview

Unsurprisingly, primary task support was the top PSD category in the analysis. Primary task principles support primary tasks, as indicated by the name. Something that was a bit surprising was that system credibility support emerged from the analysis, as it has been given less attention by both users and designers in the past. However, only 1 principle came up, and only with 3% (2/59) of the interviewees, so this was not a strong issue.

Dialogue support had 2 principles. Many of its features can be seen as supporting not only dialogue but also primary tasks. For example, *reminder* reminds the user to use a *self-monitoring* tool. Social support is another category that users may like in general.

In the analysis, only 2 social support category principles emerged from the interviews. Social features were present in the app, and they were discussed in the interviews; therefore, this result is likely related to the research question (how to mitigate hinderances) rather than a lack of interest.

#### Primary Task Support

##### Overview

*Personalization* was the top principle in this category, which was mentioned by 27 % (16/59) of the interviewees. *Tunneling* was discussed by 19% (11/59) interviewees and *self-monitoring* by 17% (10/59). *Tailoring* was brought up in 8% (5/59) of the interviews and *reduction* only in 3% (2/59) (Table 5).

**Table 5:** Primary task support features found in the analysis.

Primary task support	Interviewees (data set 1; n=29), n (%)	Interviewees (data set 2; n=30), n (%)	All interviewees (N=59), n (%)
Personalization	15 (94)	1 (6)	16 (100)
Tunneling	7 (64)	4 (36)	11 (100)
Self-monitoring	7 (70)	3 (10)	10 (100)
Tailoring	5 (100)	0 (0)	5 (100)
Reduction	2 (100)	0 (0)	2 (100)
Simulation	0 (0)	0 (0)	0 (0)
Rehearsal	0 (0)	0 (0)	0 (0)

##### Personalization

Personalization can be defined as providing personalized content or services [13]. Different ideas for personalization or even customization of reminders, menus, and content in the app emerged in the interviews. However, perhaps the most important finding regarding this theme was that all 16 (27%) interviewees felt that personalization would have improved their motivation and engagement with the app.

Given that personalization in the app only involved personalized suggestions, such as which health module to select in the beginning, it was unsurprising that the interviewees felt the app was lacking in this respect. Even “light” personalization without the participants being able to customize things would have been welcome:

Yes, so it could have taken into consideration what or who I am, my age, and the work I do and so forth. So, it would have been better if these would have been considered [in the app].

Interviewee #12

On the basis of the context analysis, many interviewees felt that the content was too general and at times felt that it was not meant for them. Personalizing the content, for example, according to the type of work done or the work environment, could reduce this issue and improve engagement. Thus, users who do not sit in front of a desk during workdays would not be encouraged to stand up regularly by the app. This type of “lighter” personalization could easily be accomplished with a few quick preuse questions (eg, “Do you work in an office environment?” or “Does your work require a lot of standing or moving?”).

The interviewees noted that many companies knew a lot about their users. For example, Google collects various data about users and their app use. The data that an app collects could then be used to personalize the app based on use patterns (eg, number of steps taken in certain periods).

Two interviewees went even further regarding their expectations of the app, suggesting self-learning algorithms:

It could be even more precise, yes it could, and I would say that artificial intelligence could be utilized, so it would match even more precisely into your own profile.

Interviewee #11

Personalization could also potentially counter the need for concurrent use of other apps or even wearables, especially if the wearables could be synced to support the app and the collected data could be used. Indeed, personalization, by providing better correspondence between the app and the needs of users, could help to remove the motivation to use complementary apps. Personalization has been shown to be effective in supporting behavior change, but it is not used to its full potential in current mHealth apps [45].

##### Tunneling

Tunneling means that the system should guide users toward the target behavior [13]. We did not implement tunneling (predesigned use paths within the app in this case) because we interpreted the *autonomy* aspect of SDT strictly. We felt that it would be best to allow users as much freedom as possible in navigation, presuming that the users would then choose the “right” actions in the app.

However, with tunneling based on personalization, the predesigned use paths could have been based on users’ own choices, thus not contradicting SDT in that sense. The same interviewee who brought up artificial intelligence regarding personalization also spoke about tunneling based on personalization:

Well, so these [use] paths, I think that they good in the sense that depending on your situation you can take a certain path [of use]. Be it exercising, or mindfulness, or [healthy] eating, or what.

Interviewee #11

Another interviewee articulated tunneling based on personalization in a more thorough manner:

Yes, a clear path which you follow so there won’t be too many options, because if you are at a crossroad and you have many paths to follow, you have to choose one, and then it may be difficult because you don’t remember which path you took. [With] one path, you can follow the tunnel to the end and only then take another, which would be so much clearer for me. When you go home and start using the app, you are like what’s the deal, but those straightforward paths take less time, when you don’t have to search [what to do next].

Interviewee #15

It became apparent from the interviews that tunneling could also “hit two birds with the same stone.” This is because many interviewees were having difficulties with both lack of time (to use the app) and information overflow (due to the broad content). Personalized tunnels would save time, as users could just start using the app even if they had only a few minutes. This is because they would not have to start by “learning” or deciding what to do next; rather, they could just go along the program until they know what to do.

Furthermore, if the tunnels or use paths are based on users’ personal preferences, users will not be overwhelmed by a massive amount of information. Instead, they will be offered only the correct path to navigate. Tunneling could also improve usability, which was problematic for many interviewees, by reducing the learning curve:

It doesn’t mean that it would necessarily have to guide you step by step, but it could repeat [for the user] the idea and what it holds, how it works, or how it should be used so you could understand. It’s the same if you have never driven a car before and you are put behind the feel with no idea or anyone saying what you must do, then it may be that you don’t succeed at the first time trying to drive.

Interviewee #7

##### Tailoring

Tailoring is related to personalization, but it focuses on user groups instead of individuals [13]. Similar to personalization, tailoring could help to address the issue of mismatching content with group levels (eg, office workers, self-employed microentrepreneurs). Moreover, tailoring could also improve users’ motivation to use the app, for example, through a social comparison function in the app (there were 2 features that showed comparisons of the results of the whole user base) that has different target groups:

It’s nice to see what kind of stress levels we micro-entrepreneurs have at certain times, but since there are so many different types of micro-entrepreneurs it is difficult to compare the results...It would have been better if there would have been like the entrepreneurs of the same line of business to check.

Interviewee #28

##### Reduction

Reduction, that is, reducing the complex behavior in the system into smaller tasks on the path to the target behavior [13], was requested by 3% (2/59) of the interviewees. Further reduction could save users some time, especially if they are extremely busy. However, as the theme only came up in 2 interviews and the app had already undergone considerable reduction at several levels (goal setting, 3 kinds of tasks from quick to long, and easy-to-use tools), further reduction would likely have only a minor effect on improving user engagement.

With other apps, it might be more beneficial for the “medicine” for behavior change to be provided in “doses.” This would be especially useful if the users are busy, tired, or stressed as by focusing on small steps, designers can avoid overloading users’ cognition.

##### Self-Monitoring

Self-monitoring means that the system should provide a means for tracking one’s performance or status [13]. The app provided several types of self-monitoring, including self-reporting levels of stress or recovery and a variety of tools (eg, an alarm to remind the user to stand up). However, there was only one self-monitoring tool that took advantage of smartphone sensors for “automatic” measurement, a step counter. On the basis of the interviewees’ statements, improving the “automatic” monitoring functions of the app (via sensors or even syncing external wearables) could help to increase user engagement:

I thought that this app would remind me about it [going to bed], and via the app I could also measure like an engineer what it actually is [amount of sleep], so it wouldn’t just be gut feeling [how much I sleep]. In a way, it would be a motivator, that kind of monitoring tool, which would help me to see the direction I’m going to and do I have some difficulties regarding sleeping or not.

Interviewee #29

Technostress can be mitigated by controlling the way technology is used and by distancing oneself from technology use when feeling stressed [46,47]. By enabling automated self-monitoring (via sensors) or syncing wearables to the app, designers could actively reduce technostress for users.

Overall, 29% (17/59) of the interviewees reported symptoms of technostress, and 5 (29%) of them stopped using the app. This is an important issue that would also affect similar apps. Thus, mitigating technostress could have an important impact on engagement. This is especially important for target groups that use technology as a means of working—they may not want to use mHealth apps to recover from work or manage stress if the app use reminds them of their work:

It should have had, well something like activity bracelet or other automation. For me, it proved out to be too big of an issue to type things on my smartphone, because then I get the feeling that I must do that too much already, so I just want to get rid of that [typing on smartphone].

Interviewee #42

#### Dialogue Support

##### Overview

In 19% (11/59) of the interviews, the interviewees’ opinions about dialogue support focused mostly on *reminders*. Three interviewees (5%) also brought up *liking* (Table 6).

**Table 6:** Dialogue support features found in the analysis.

Dialogue support	Interviewees (data set 1; n=29), n (%)	Interviewees (data set 2; n=30), n (%)	All interviewees (N=59), n (%)
Reminders	9 (82)	2 (18)	11 (100)
Liking	2 (67)	1 (33)	3 (100)
Praise	0 (0)	0 (0)	0 (0)
Rewards	0 (0)	0 (0)	0 (0)
Suggestion	0 (0)	0 (0)	0 (0)
Similarity	0 (0)	0 (0)	0 (0)
Social role	0 (0)	0 (0)	0 (0)

##### Reminders

Reminders from the system can remind users of their target behavior when using the system [13]. The interviewees felt that reminders should be meaningful and even customizable, which in this paper is linked to personalization. One interviewee (2%) thought that only getting a weekly reminder would be sufficient, whereas 2 (3%) interviewees proposed a weekly reminder in the form of a weekly review in addition to other reminders:

I would like this to be more active, it should be more active for the users in some way. Weekly review would be very good, or weekly reminder on it, then it would work really well.

Interviewee #2

Two (3%) interviewees indicated that they would have been satisfied with fewer reminders, whereas 15% (9/59) of the interviewees wished for more than they had received:

It [low use] is partially because I didn’t realize how good it is [the app], so maybe in the beginning there should have been [more] reminders. Naturally, some may be irritated by those, if some program reminds that now you have taken 10,000 steps, but this could have reminders more like think about this or have you checked that.

Interviewee #8

It should be mentioned that the push notifications in the app did not work perfectly for all users, which could have affected why 15% (9/59) of the interviewees requested more reminders. However, to the best of our knowledge, the bug was caused by mismatched libraries owing to the different Android versions. Therefore, it only applied to certain timed weekly reminders and not all reminders. Those that could have been triggered by choosing longer tasks seemed to work better according to the use log data.

##### Liking

Liking implies that the look and feel of the system should appeal to users [13]. A multitude of visually attractive pictures were used, and attention was given to how the text was set up and sectioned in the app. However, according to 5% (3/59) of the interviewees, the same principle was not applied to all the infographs in the app:

When some graphs like in the app comes along, for me these are like something that I bypass very easily, since I just think that I don’t understand these kinds of crooked objects, or I don’t want to concentrate on them.

Interviewee #15

Improving the visual design of the infographs—making them easier and clearer to perceive and understand—would likely help to solve some issues regarding usability and content. In general, if users have difficulty in understanding or even noticing some aspects of the app, they will use the app, or at least those aspects of it, less.

One user even mentioned during the interview that they had used the tool with an infograph but had not paid much attention to it. A graphic designer worked on other parts of the app, but in hindsight, she should have also checked the infograph designs before implementation.

#### System Credibility Support

##### Overview

Only 3% (2/59) of the interviewees mentioned principle or principles related to system credibility support (Table 7), which was unsurprising. Features in this category are more difficult to implement as distinct technical features in apps. Some of them are even concepts that people have grown accustomed to and assume to be part of every app. For example, the expertise principle states that mobile apps should be updated regularly [13], which is something that is performed in the background.

Therefore, the lack of mention of these principles in this analysis does not mean that they are not good features; rather, they were already present to a sufficient degree. For example, some users mentioned that having the Finnish Institute of Occupational Health involved was important, which could be linked to expertise or even authority and trustworthiness depending on user’s perspective.

**Table 7:** System credibility support features identified in the analysis.

System credibility support	Interviewees (data set 1; n=29), n (%)	Interviewees (data set 2; n=30), n (%)	All interviewees (N=59), n (%)
Real-world feel	0 (0)	2 (100)	2 (100)
Trustworthiness	0 (0)	0 (0)	0 (0)
Expertise	0 (0)	0 (0)	0 (0)
Surface credibility	0 (0)	0 (0)	0 (0)
Authority	0 (0)	0 (0)	0 (0)
Third-party endorsements	0 (0)	0 (0)	0 (0)
Verifiability	0 (0)	0 (0)	0 (0)

##### Real-World Feel

Real-word feel can make it possible to contact specific people through the system [13]. In total, 3% (2/59) of the interviewees brought up a real-world feel as a potential improvement to the app. One of them even stopped using the app due to receiving face-to-face guidance in real world. We agree that mHealth apps could use real-word feel, for example, through chats or meetings with health personnel, or as one of the interviewees expressed it:

It could be for example a nurse who you would meet regularly so you would follow [your progress] together with the nurse and you would be moving forward [towards personal goal]. So, a continuous care or well-being relationship would be formed. Yes, something along that line.

Interviewee #54

For users struggling with difficulties related to guidance (different coaching preferences), real-world feel in the app could improve engagement. However, it is unclear how realistic it would be to implement real-world feel in meetings (either “live” or internet based) if the purpose is to develop cost-efficient health intervention apps. Such a feature might work better with more specialized or highly commercial (pay-per-use) guidance apps.

#### Social Support

##### Overview

Overall, 5% (3/59) of the interviewees mentioned social learning. Two discussed social comparison, which already existed as a feature in the app (Table 8).

**Table 8:** Social support features identified in the analysis.

Social support	Interviewees (data set 1; n=29), n (%)	Interviewees (data set 2; n=30), n (%)	All interviewees (N=59), n (%)
Social learning	3 (100)	0 (0)	3 (100)
Social comparison	2 (100)	0 (0)	2 (100)
Normative influence	0 (0)	0 (0)	0 (0)
Social facilitation	0 (0)	0 (0)	0 (0)
Cooperation	0 (0)	0 (0)	0 (0)
Competition	0 (0)	0 (0)	0 (0)
Recognition	0 (0)	0 (0)	0 (0)

##### Social Learning

Social learning suggests that users will be more motivated if they can observe others engaging in similar behaviors via the system [13]. Overall, 5% (3/59) of the interviewees indicated a desire for a networking or social learning feature in the app, if nothing else, at least peer chat support. The participants in our target group seemed to be social or at least interested in networking. This may be because networking may lead to business opportunities, and one can safely let off the steam caused by entrepreneurship with peers:

For micro-entrepreneurs, self-employed entrepreneurs, or those who employ few people, they may have little connections or networks. For these kinds of people, they could use this kind of [feature in the app]. I don’t know, perhaps chat, so they could share things safely among themselves.

Interview #24

Sharing issues, such as worries or job-related strains, allows users to see that they are not alone, and in the case of the trial app, to see that others are working on recovering from work as well as trying to change their behavior in a healthier direction. Enabling social learning (or even networking) could resolve issues related to the “expectations unfulfilled” theme, and the interviewees stated that networking or peer support should be a part of the app. It is possible that this feature could reduce the obstacles for some users regarding use, but the interviews did not provide enough data to determine whether this is an important issue with this type of health app in general.

##### Social Comparison

Social comparison enables users to share and compare meaningful information with other users, which can increase their motivation to perform the target behavior [13]. The app had 2 social comparison features, but they targeted the entire user base. Overall, 3% (2/59) of the interviewees stated that this feature was not useful because of the lack of distinct groups:

Well, it didn’t [influence me] because there are probably some many different kinds of people, so at least I couldn’t see a distinct trend from it [social comparison feature].

Interviewee #4

With tailoring, it could be possible to divide users into different groups and only show comparison data from the group that equates to user. This could increase motivation for some, but it is unclear whether this would address any of the themes found to hinder their use in this study.

## Discussion

### Principal Findings

This paper presents some unanticipated findings, but in hindsight they are logical. They also showed that background theories should not be interpreted too strictly, or at least designers should find ways around them.

For example, when designing the app, we felt that we could not use *tunneling*, a PSD feature that “guides” users via a path toward the desired behavior. This is because SDT was used as the background theory, and there was concern that tunneling might interfere with the “free will” (autonomy) of the users. Thus, in seeking to avoid restricting the users, we managed to alienate the users who wanted “tour guidance” in using the app and their behavior change process.

### Dual Role of Microentrepreneurs

The microentrepreneurs in this study appear to play dual roles. They were representatives of their own business, which affected the specific work-related strains and stressors they encountered and thus their recovery from work. At the same time, they were entrepreneurs, resulting in another range of strains and stressors, especially for those with employees. The dual roles of target groups represent a design challenge. From this viewpoint, will they use the apps? Do they have time to use apps at all?

Because of these dual roles, it was not surprising that two-thirds (42/59, 71%) of the interviewees reported being very busy, which seemed to be characteristic of their lives in general. When planning a trial for people with dual roles (or designing apps for them), it is important to consider that they may not be willing to spend a lot of their time. They may already be busy with other tasks and have no time for anything “extra.”

### Need for Time-Saving Guidance

Given that many interviewees reported being busy, the need for better and personalized guidance was evident from the interviews. It is logical that busy people would like to avoid learning curves (with the help of tunneling) and only spend time on things that are explicitly useful to them (with the help of personalization).

Using *tunneling*, *tailoring*, *reduction*, and *personalization* may improve engagement as each principle can involve time-saving elements in the right context. With *tunneling*, the path is laid out for the user, especially when combined with *personalization*, and thus the learning curve should be less steep. Furthermore, users are not required to decide what to do in the app; instead, they can follow the guidance based on personal preferences.

In addition, if *reduction* is used correctly, users can digest small bits of information when they have time and do not become stressed due to lack of time. Moreover, reduction can save users from trying to absorb the whole thing at once, which may lead to information overflow and dropping out from the guidance program. *Personalization* could also enhance the user experience, as it would allow personalized content, which would certainly be more meaningful than general information for the user.

### Technostress

Overall, 8% (5/59) of the interviewees reported experiencing technostress during the trial and quit using the app. Although this was not a common occurrence, they quit the trial due to technostress. In addition, technostress manifested when users had to learn how to use the app, although it did not require an insurmountable effort.

We learned from the analysis that interviewees who were already stressed due to work did not like using the same platforms or devices for recovery that they also used for work. Self-monitoring tools that are synced to wearables (or that use smartphone sensors for automatic measurement) could help users who want to take measurements while distancing themselves from active smartphone use outside of office time to decrease technostress.

Learning to use the app required at least some effort to read the instructions, which might have been too much for some users, particularly if they were already exhausted. Therefore, due to the lack of time and job-related strain, this may have triggered further technostress in some interviewees. Furthermore, technostress was likely increased by the *reminders*, as the participants could not customize (*personalize*) them. They could only turn off the reminders for each task after receiving the first push notification.

### PSD Postulates

The sixth PSD postulate states that a system should not be obtrusive; in this case, *personalization* could have decreased the obtrusiveness of the app. Obtrusiveness was caused by reminders triggering at the wrong time (when the interviewees could not react to them because they were working). Therefore, designers should enable customization of push notifications in systems—or at a minimum the ability to turn them off.

The fifth PSD postulate emphasizes transparency. Accordingly, designers should disclose what their apps are based on. Some interviewees stated that they knew that there was the Finnish Institute of Occupational Health behind the app, and so they felt it was trustworthy. Thus, there should not have been any confusion about the app being used for research and that it was not a commercial one. Nevertheless, it seems that we could have done better in informing people enrolled in the trial, as some interviewees clearly did not realize what they had enrolled in or presumed they would be using apps similar to commercial ones. This could reduce use, so designer bias (the app being for research) should be clearly disclosed to users to avoid confusion.

Regarding whether to use direct or indirect persuasion (the third PSD postulate), it seems clear that in the case of microentrepreneurs, the indirect approach is better. The participants were constantly busy with their work; two-thirds (42/59, 71%) of the interviewees did not seem to have enough time to use the system, which also meant that the persuasion process might not have affected them continually or even incrementally as intended (first and fourth PSD postulates).

In addition, information overflow seemed to be an issue, as the interviewees reported that they had difficulties deciding on what to choose within the app. Thus, an indirect approach might be better if there are several possible ways to use an app or if several health problem domains are addressed in a single app. Moreover, we recommend using the *tunneling* principle in similar cases, as it could help the users with the learning curve and save precious time, thus enabling the system to be more open (“always on”) and the persuasion process to be incremental because users actually use the system. In addition, this could increase users’ commitment (second PSD postulate) by making it easier to use the app. Intuitively, it is easier to commit to something that can be used with a “plug-and-play” mindset rather than something that requires a steep learning curve.

The information overflow and the steep learning curve reported by the interviewees might have partially been a result of our strict interpretation of the autonomy aspect of SDT, which led to giving the users excessive freedom when navigating the app. If we had (better) used *personalization*, the choices for “*tunnels*” or use paths could have been the users’ own, in which case there should be no contradictions with SDT.

The seventh postulate of PSD encourages the design of useful and easy-to-use apps. Therefore, researchers and designers should be realistic about the features and the content of mHealth apps. For example, small start-ups or smaller research projects may not have adequate resources to implement everything. Carefully drawn lines defining what can and cannot be done with the given resources would result in more stable apps and fewer bugs for users (or developers) to worry about.

### Lessons Learned

We acknowledge that the usability of the app could have been improved, as is evident from the analysis. In addition to usability issues, bugs in the app also reduced use. Although half (30/59, 51%) of the interviewees reported encountering bugs, most of them did not contact our helpdesk for technical support. Apparently, providing technical support via email alone is not sufficient for bug reporting [48]. It should be noted that some of the bugs reported by the interviewees could have been usability issues rather than technical difficulties.

The expectations of the users regarding the app seemed to be at least partially based on commercial health apps, and some were even mentioned during the interviews. Commercial apps differ from the app used in the intervention. The contents of the intervention app were evidence based, and the app was based on behavior change theories. At least in part, this could explain why some people felt that the tasks were different than those of commercial apps. Furthermore, based on the interviews, people have become accustomed to commercial apps having bugs, which are fixed eventually.

When enrolling in the intervention, not all participants may have had clear personal goals. Some may have joined simply out of curiosity, wanting to test the app. If it did not seem to suit their needs immediately or they felt it was too complex, they might have just abandoned it and moved on to the next one, and there are plenty available in the commercial market. Therefore, there is no need to try to engage 100% of users, as some people may just want to test it and may not be ready to engage.

We also recognize that it is not always an easy task to prioritize features in the design phase, and target users may end up behaving differently regarding app use than the designers originally predicted. Therefore, it is important to increase the knowledge about different user groups. However, it is not practical to try to meet every imaginable need of users, as there will always be some who will not be happy. Indeed, trying to fit everything into a single app may lead to poor design or imperfect implementation, thus benefitting no one.

### Limitations

The limitations of this study include the differences between the data sets, as the 2 teams conducting the interviews used different sets of semistructured questions. The emphasis of the interviews was also different between the teams, although the themes of the actual questions overlapped in both data sets.

The results can be generalized to similar groups to a certain extent, and a persuasive event analysis would be helpful for identifying those groups. However, it is also possible that different results could be obtained with similar groups.

The thematic analysis process was conducted with utmost care to identify all the sources relevant to the themes that emerged. Regarding the study and app use, the Hawthorne effect [49] was considered one of the potential themes in the first analysis, as some users brought up the study setting in the interviews. They were conscious of the ongoing research as they had enrolled in it themselves, and thus, they might have felt a responsibility to use the app. However, it was not possible to determine whether the Hawthorne effect increased or decreased the app use. Therefore, this theme was removed from the analysis.

### Declaration of Bias

To avoid researcher bias in the interviews, the interviewees were encouraged to answer the questions frankly and sincerely, and they were assured that there were no right or wrong answers. Interviewers from both teams also tried to avoid any steering of the interviewees in any direction.

Although we cannot be completely certain that the interviewees’ responses fully portrayed their experiences, we trust that they attempted to answer the questions as honestly and sincerely as possible. This trust was further enhanced by the fact that both positive and negative experiences were discussed during the interviews by all interviewees. In addition, there were different emphases in the question sets used by the different interview teams, which helped to mitigate any unintentional bias in the whole data set. The data used in the analysis were obtained from 2 different research teams and 2 different data sets.

### Conclusions

It is important to “know your audience” to predict the potential factors that could hinder use, as it is easier to deal with those factors up front. Some of these factors could be avoided entirely, especially those linked to the design of the system.

Factors associated with the users could be harder to avoid, especially if they are not recognized beforehand. Many of the factors presented in this paper may seem somewhat universal, such as being busy due to work or the bug types found in the system. However, there are other factors that are much harder, perhaps impossible, to counter, such as negative situations in users’ lives (eg, noisy neighbors or the death of a family member).

The PSD postulates present logical aspects and concerns for designing persuasive or other types of systems. However, it may not always be easy to apply them in practice if time and resources are scarce. No one wants to build flawed or buggy systems, but even so, many information systems projects fail.

This is a universal problem, and it comes down to the 3 well-known constraints of the project management triangle and system quality: cost, time, and scope. It is not possible to change only one constraint without affecting quality. Therefore, if the scope, cost, and time are not balanced, it will be challenging to build persuasive (or any other) systems.

Persuasive principles are tools in the design toolbox that can motivate and engage users to strive for behavior change, and in the best-case scenario, they lead to support systems becoming obsolete because users reach their personal goals. However, the persuasive principles are not silver bullets. Careful consideration is required in terms of when and how they should be used. It is crucial to “know the audience,” so the right tools can be selected from the toolbox and put into use to support the users of the designed system in their behavioral change processes.

### Implications

Our paper has the following implications:

1. This paper increases knowledge regarding microentrepreneurs, which can be generalized to people with dual roles for example in terms of work and study. Increasing current knowledge about target groups for persuasive design is vital, as studies on this subject are rare. Persuasive design seeks to motivate and engage users to use systems, but limited knowledge about target groups can lead to decreased persuasion. Conversely, increased knowledge could lead to better opportunities to persuade users.
2. Drawing on the PSD model, the paper proposes context-specific solutions to several issues that hinder or reduce the use of similar systems. However, we acknowledge that everything cannot be “designed away.”
3. The paper discusses the role of PSD postulates in improving systems, which has implications for both researchers and designers. Moreover, this paper contributes to the knowledge on how the postulates can be used or aligned for both research and design.
4. This paper also presents a PSD-based solution for a “strict” interpretation (of the autonomy aspect) of SDT in terms of navigation and user freedom. Through the use of personalized tunneling, it should be possible to provide use paths for users based on their own choices, thus not contradicting SDT.
5. We believe that this paper can function as an example of how to use thematic analysis to (1) increase knowledge on target groups through inductive analysis and (2) find theory-based solutions for issues through deductive analysis.
6. We have demonstrated one way to tie inductive thematic analysis with theory commitment, in this case, with persuasion event contexts from the PSD model. Persuasion event context analyses are not common, and thus this paper provides an important example of using such an analysis in research.

### Future Research

Other target groups with similar issues should be studied in the context of persuasive mHealth apps to uncover similarities or differences between different groups. This would help to generalize the findings regarding persuasive mHealth apps. Future studies could also examine personalized tunneling in terms of app use engagement, which could be helpful for many users.

## Appendix

Multimedia Appendix 1 Question set 1.

Multimedia Appendix 2 Question set 2.

## Abbreviations

mHealth: mobile health
PSD: Persuasive Systems Design
SDT: self-determination theory
SME: small and medium-sized company
TTM: transtheoretical model
