# Supplementary material for: Factors Reducing the Use of a Persuasive mHealth App and How to Mitigate Them: Thematic Analysis
Source: JMIR Hum Factors. 2023 Jun 26;10:e40579. doi: 10.2196/40579 (PMC10337416; doi:10.2196/40579)
Supplement: Multimedia Appendix 1 [file humanfactors_v10i1e40579_app1.pdf]

1. Where did you originally learn from this guidance program / smartphone app?
2. What has it been like to be a part of this program? How was it when you were using the app?
3. What kind of goal did you set for yourself in the beginning?
4. What were the features in the app that helped you to achieve your goals?
5. What made you use the smartphone app? / What made you to stop using the smartphone app?
6. How did the app fit into your everyday life? Did you have enough time to use the app?
7. How credible was the app in your opinion and what were the issues that had the most impact on the credibility?
8. What do think about the app and yourself as the user of the app:
    a. I see myself as an experienced user of mobile services.
    b. This mobile service gives me suitable challenges.
    c. I often feel insecure when using this mobile service.
    d. I can learn new things with the help of this mobile service.
    e. When using this mobile service, I feel completely engaged into what I'm doing.
    f. I think that the structure and the whole mobile service is easy to understand.
    g. Navigating in this mobile service is easy.
    h. I think that it's easy to use this mobile service.
    i. I think that this mobile service is beneficial to me.
    j. I think that using this mobile service is pleasant.
9. What did you think about the messages received from the app? How often would you like to receive messages and other feedback from the app?
10. Do you think that the information received via the app is personal enough?
11. It is possible to share experiences with others in the app. Do you use or follow the feature?
12. When thinking about the previous question, why do you use/follow the feature or don't use/follow the feature (depending on previous answer)?
13. How convincing this app is in your opinion? Does it persuade you? Does it make you reach towards your target goals?
14. What would it take for the system to be more engaging?
15. Has the app managed to entertain you, or do you consider it to be explicitly for benefit?
16. What if the app would be emphasized more towards being entertaining?
17. Does using the app feel easy?
18. Does the app make you to adhere to the program, has it changed your behavior and has it influenced your attitudes?
19. Does the app provide you enough challenges?
20. Have you been motivated during the guidance program?
21. Please comment the situation regarding changes in your ways of acting:
    a. I don't think I have to change my ways of acting.
    b. I have noticed a need to change my ways of acting.
    c. I intend to change my ways of acting.
    d. I have already consciously altered some of my ways of acting.
    e. I have managed to act in a new way for some time now.
22. Have these changes been noticeable in your well-being?
23. Have there been any unplanned negative consequences from using the app?
24. Do you also have a goal for the future? Is it the same as in the beginning when you started using the app or has it changed in some way? Do you think that you will reach your goal?
25. Can you think of something that I should have asked you during this interview or do you want to comment something (free to comment anything)?
