# Supplementary material for: Factors Reducing the Use of a Persuasive mHealth App and How to Mitigate Them: Thematic Analysis
Source: JMIR Hum Factors. 2023 Jun 26;10:e40579. doi: 10.2196/40579 (PMC10337416; doi:10.2196/40579)
Supplement: Multimedia Appendix 2 [file humanfactors_v10i1e40579_app2.pdf]

1. **How did you end up being an entrepreneur?**
   a. Describe your path towards entrepreneurship?
   b. How would you characterize yourself as an entrepreneur?
2. **How would you describe the current situation of you being an entrepreneur?**
   a. What is the situation of your company right now? How do you see the future? Do you want to continue as an entrepreneur, or do you miss something from working as paid labor?
   b. How profitable is your company? How well do you get along with the profit from your company? What does it mean to you, that your company is financially profitable? How do you see your health in relation to your business?
3. **What is the best thing about being an entrepreneur and what is the most difficult thing?**
   a. How do these things affect the way you see yourself and what kind of perception do you have regarding yourself as an entrepreneur
4. **How do you take care of yourself (nutrition, sleep, exercise)? What does health mean to you and what are the things it consists of? What kind of issues affect your way of living?**
   a. What effect does entrepreneurship have on recovery from work, way of living, work ability?
   b. What does work ability and health mean to you as an entrepreneur?
   c. What are your means for recovery? What and what kind of things affect your recovery? What are your means for recovery from work?
   d. How does your own ordinary days of life differ from the most optimal way of supporting health and recovery from work?
   e. Why doesn't this "quiet knowledge" realize in everyday life? How would you explain this phenomenon?
   f. What would support this "quiet knowledge" to actualize?
   g. As an entrepreneur, what kind of things are you able to stand in your work?
   h. What fields of health are realized and what are not? Why? What is the cornerstone of having enough strength to go on?
5. **Guidance program via smartphone app – recovery from work and work ability**
   a. Where did you learn about this research? What kind of thoughts did you have about it? What kind of things made you to come along?
   b. How did the smartphone delivered guidance program affect your thinking?
   c. behavior?
   d. Did you change your health behavior and if so, how?
   e. How do these changes in behavior show up in your enterprise/work/everyday life?
   f. How did the contents in the application support changes?
   g. What made you to change your behavior, what inspired you? What didn't help at all?

6. **Receiving support (from who or via what?)**
   a. recovery from work?
   b. health behavior?
   c. changing ways of life?

7. **Do you have occupational health care contract? What kind of support have you received from occupational health care? If you don't have a contract, why is that? If you do have a contract, how do you think the services of occupational health care match the needs of entrepreneurs?**

8. **What kind of occupational health care services should be developed for entrepreneurs?**

9. **Has entrepreneurship changed you?**
    a. if it has, could you describe how?
    b. What kind of change has there been (for example) between being paid labor and being an entrepreneur?
    c. Has there been changes in how you see yourself?
    d. Could you talk about your health and health behavior regarding your life. Have you changed your way of life? What are your experiences regarding your health now and what do you think will happen in the future?
10. **Could you tell what kind of people are other entrepreneurs and what is a typical Finnish microentrepreneur? What kind of behavior do you think other entrepreneurs have regarding health?**
    a. How would you describe those entrepreneurs that you know?
    b. Do you think entrepreneurs have common attributes between themselves?
    c. Where do these attributes come from?
    d. Do other entrepreneurs think similarly regarding entrepreneurship? What do you think, do other entrepreneurs think similarly to you?
11. **Do the opinions regarding yourself differ from how the society perceives entrepreneurs?**
    a. What kind of stereotypes others have regarding entrepreneurs?
    b. How do entrepreneurs deal with these stereotypes?
    c. Can anyone else understand entrepreneurs than entrepreneurs themself?
12. **What kind of social networks do you have and how do communality and social networks affect health behavior choices?**
    a. What do entrepreneur colleagues and social networks mean to you?
    b. What kind of issues do you discuss with colleagues, if you see them away from work?
    c. Do you participate in communal meetings between entrepreneurs?
    d. What kind of meaning do these meetings have in regards building social networks between entrepreneurs?
    e. What kind of feelings originate in these meetings?
13. **How do other entrepreneurs understand relevance of health and health behavior regarding recovery from work and work ability?**
    a. how do entrepreneurs discuss health and health behavior between themselves?
    b. how do entrepreneurs discuss mental resilience and the burden of entrepreneurship between themselves?
    c. Are there any shared events, where recovery from work and work ability are supported?
    d. What kind of meaning do you think communality has regarding recovery from work and work ability?
